# How Many Types of Dystonia? Pathophysiological Considerations

**DOI:** 10.3389/fneur.2018.00012

**Published:** 2018-02-23

**Authors:** Angelo Quartarone, Diane Ruge

**Affiliations:** ^1^Department of Biomedical, Dental Sciences and Morphological and Functional Images, University of Messina, Messina, Italy; ^2^IRCCS Centro Neurolesi “Bonino Pulejo”, Messina, Italy; ^3^Department of Psychology and Neurosciences, Leibniz Research Centre for Working Environment and Human Factors, Technical University Dortmund, Dortmund, Germany

**Keywords:** dystonia, plasticity, TMS, tDCS, basal ganglia

## Abstract

Dystonia can be seen in a number of different phenotypes that may arise from different etiologies. The pathophysiological substrate of dystonia is related to three lines of research. The first postulate a loss of inhibition which may account for the excess of movement and for the overflow phenomena. A second abnormality is sensory dysfunction which is related to the mild sensory complaints in patients with focal dystonias and may be responsible for some of the motor dysfunction. Finally, there are strong pieces of evidence from animal and human studies suggesting that alterations of synaptic plasticity characterized by a disruption of homeostatic plasticity, with a prevailing facilitation of synaptic potentiation may play a pivotal role in primary dystonia. These working hypotheses have been generalized in all form of dystonia. On the other hand, several pieces of evidence now suggest that the pathophysiology may be slightly different in the different types of dystonia. Therefore, in the present review, we would like to discuss the neural mechanisms underlying the different forms of dystonia to disentangle the different weight and role of environmental and predisposing factors.

## Introduction

Dystonia is defined as a “movement disorder characterized by sustained or intermittent muscle contractions causing abnormal, often repetitive, movements, postures, or both” (1). Dystonic movements are typically patterned, twisting and may be tremulous. Dystonia is often triggered or worsened by voluntary action and is typically associated with overflow muscle activation (1). Dystonia may have different etiologies and present with different phenotypes (2). Several lines of research have generalized the pathophysiology of one type of dystonia to all types (2). On the other hand, given the extreme heterogeneity of the different phenotypes it is plausible to assume that there are different pathophysiological substrates where environmental factors and genetic factors may play a different weight and role.

Therefore, in the present review, we would like to provide a critical reappraisal of the pathophysiology in the different forms of dystonia.

## Pathophysiology of Dystonia: General Consideration

There are at least three general themes that have emerged from research around dystonia. First, it is well known from lesion studies that dystonia can be caused by damage to multiple brain regions such as basal ganglia (BG) (often seen in imaging studies of idiopathic dystonia as well), but also the thalamus, brainstem, parietal lobe, and cerebellum (3).

Indeed, although there is no evidence of neurodegeneration in idiopathic dystonia, a variety of subtle microstructural and functional abnormalities have been reported. In particular, several structural and functional neuroimaging studies have revealed extensive functional and structural abnormalities involving several brain regions in keeping with the idea that dystonia is a network disorder (4–7). Second, due to the lack of apparent neural damage in idiopathic dystonias, another line of research postulates that dystonia may be included in the category of neuro-functional disorders, which arise from subtle abnormalities of inhibition and sensory-motor integration (6).

The lack of inhibition across multiple level of the central nervous system may be responsible for the excess of movement and for the overflow phenomena seen in dystonia (6).

In addition, although dystonia is generally regarded as a pure motor disorder, another major theme in the pathophysiology of dystonia is a defect in sensory or perceptual function or in “sensorimotor integration.”

Patients with focal dystonia have difficulty in discriminating sensory stimuli in both spatial and temporal domains (8). In addition, sensory modulation in response to movement, the so-called sensory gating, is abnormal in focal hand dystonia (FHD) (9). Finally, the third block of research postulates that during motor learning the mechanisms of neuroplasticity are abnormal. Indeed, when we learn a new motor skill, the presence of flexible, plastic changes within neural circuits allows a fast adaptation to a dynamic environment, thus, facilitating learning and memory.

These dynamic plastic mechanisms need to be strictly bounded to avoid excessive change and synaptic destabilization, a phenomenon called homeostatic plasticity. This fine regulation of plasticity is deranged in dystonia producing a maladaptive plasticity (see below).

This unconstrained plasticity may explain why, in focal dystonias, environmental factors, such as repetitive training or peripheral nervous system injury, may lead to uncontrolled reorganization of sensorimotor maps and the eventual development of dystonic symptoms. Finally, in recent years, advances in sequencing technology have boosted up the discovery of new genes that appear to be relevant in dystonia.

The discovery of new causative genes is the first step to disclose the complex molecular pathophysiology in familial but also in sporadic forms of dystonia and to better understand the alterations at system level.

## Task-Specific FHD

Focal hand dystonia is the result of a combination of an abnormal sensory–motor plasticity and abnormal inhibition along with environmental factors such as intensive training (10). Alterations of inhibitory circuits have been widely reported across central nervous system in dystonia (11). Although these findings are not specific for dystonia, abnormal intracortical mechanisms have been reported in both hemispheres despite unilateral symptoms and even in asymptomatic body regions (12). For this reason, there is not a direct link between reduced intracortical inhibition and dystonia.

On the other hand, the lack of inhibition may contribute to the typical dystonic unfocussed muscular activation.

It has been postulated that surround inhibition is an operating system with motor areas producing a more accurate movement, just as surround inhibition in sensory systems allows a more exact perception (13).

Surround inhibition is reduced in FHD and this may contribute to the difficulty in focusing motor command and to overflow phenomena and may determine the excess of plasticity (14).

An important clinical feature is that typically hand dystonia is triggered by a period of intensive training of skilled movements.

The role of overtraining in dystonia has been corroborated by animal studies in primates. Indeed, experimental evidence in monkeys suggests that a relatively short period of overtraining may subvert the connectivity of sensory and motor cortices leading to an inappropriate integration between sensory input and motor outputs, which could culminate in overt dystonia (15).

Although it is reasonable to assume that intensive training could potentially lead to abnormal reorganization of the sensorimotor cortex, such as in musicians, producing the classic cramp, on the other hand, it is not as clear why only some subjects develop dystonia during intensive practice.

**Figure 1:**
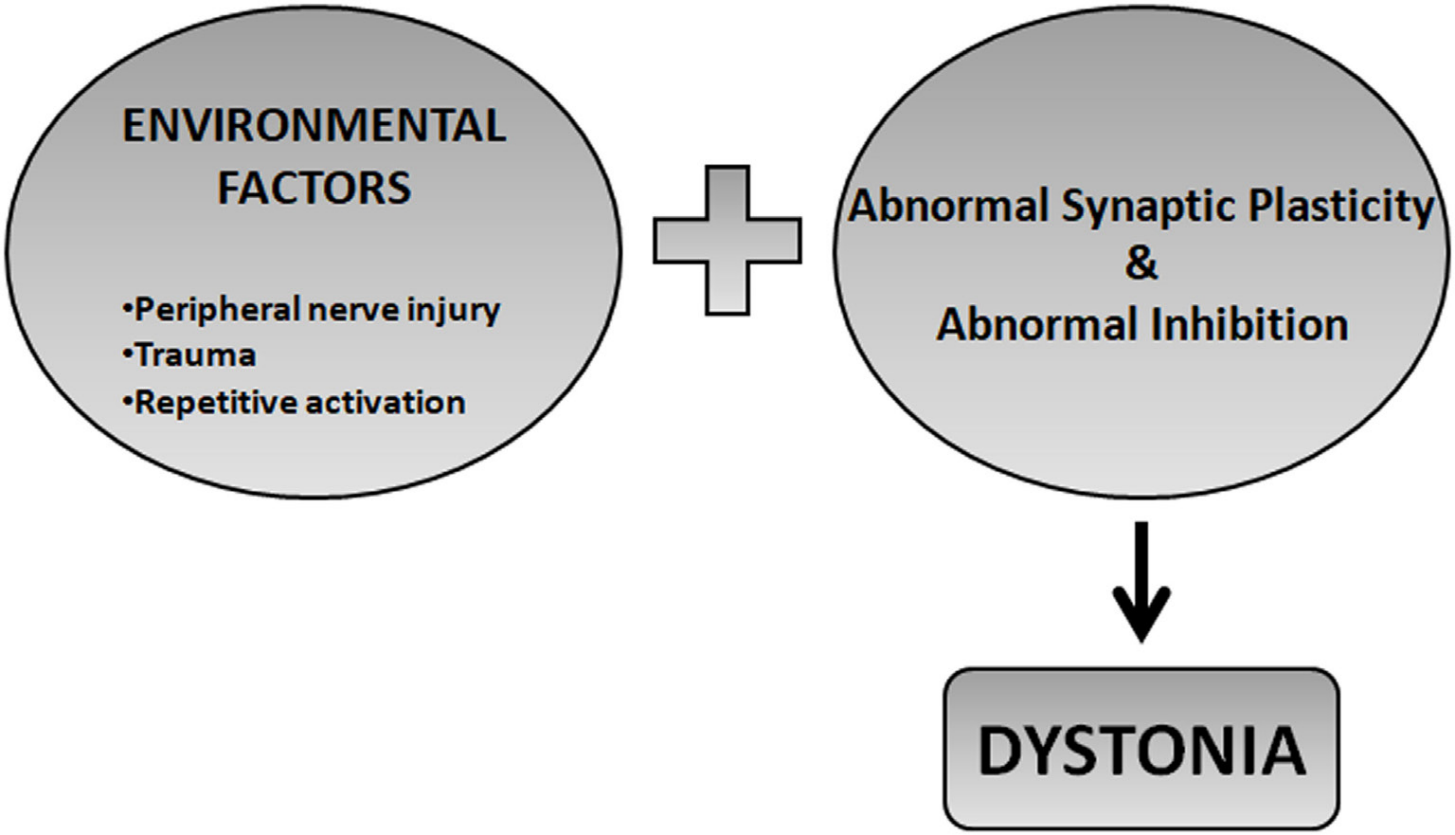
Two factor hypothesis. FHD may develop in predisposed individuals where subtle abnormalities of plasticity may, in conjunction with repetitive hand training or other environmental factors, trigger the development of dystonic postures.

**Figure 2:**
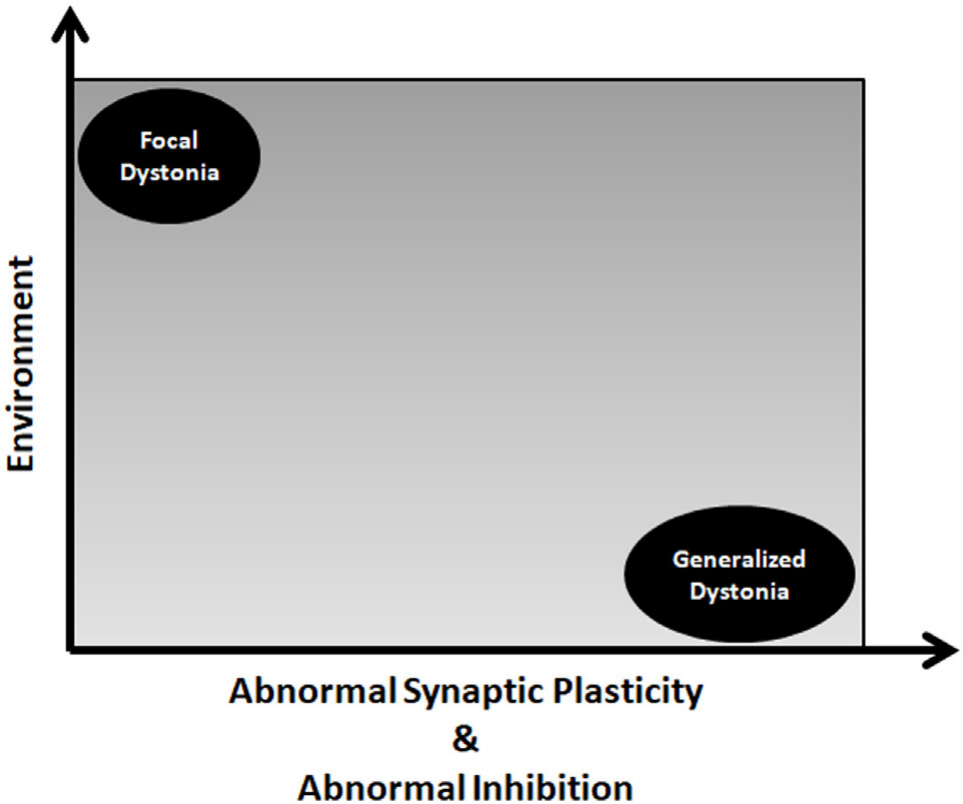
Impact of two factor hypothesis in dystonia spectrum. The role of environmental factors is less important in triggering generalized dystonias where important structural plasticity changes may take place.

The two factor hypothesis postulates that FHD may develop in predisposed individuals where subtle abnormalities of plasticity may, in conjunction with repetitive hand training or other environmental factors, trigger the development of dystonic postures (Figure 1). This is intuitive for occupational dystonia such as musician’s cramp and writer’s cramp where dystonic postures develop if plastic changes are pushed to their extreme by repetitive movements (10). The role of environmental factors is less important in triggering generalized dystonias where important structural plasticity changes may take place (Figure 2).

In line with this hypothesis, several groups have demonstrated that sensory–motor associative plasticity, tested with transcranial magnetic stimulation (TMS), is enhanced in patients with FHD and also in patients with cranio-cervical dystonias representing an important endophenotypic trait (16–20).

This is also supported by the normal responsivity of sensory–motor cortex to paired associative stimulation (PAS) in patients with secondary dystonia (21) suggesting that the abnormal plasticity is not the mere consequence of the abnormal posture but rather an idiopathic, causative abnormality.

Despite the altered responsiveness of sensory–motor cortex to TMS that has been reported by many studies, considering the relatively small sample and different methodologies employed, it would be important, in the future, to launch large multicentre studies to better explore the variability of sensory–motor plasticity in primary dystonia (22).

In addition, it is likely that the functional abnormalities of plasticity may translate into structural abnormalities as suggested by a recent study showing extensive gray matter and white matter changes in patients with task-specific dystonia (23).

If abnormal plasticity is a predisposing factor to develop dystonia, then it is mandatory to understand what pushes plasticity beyond its physiological boundaries in patients with focal dystonia. The homeostatic plasticity regulation is an essential prerequisite in nature to keep overall synaptic weight in neuronal networks within a useful physiological range (24).

In keeping with this theory, there are very strict homeostatic rules that prevent an uncontrolled increase in synaptic effectiveness produced by long-term potentiation (LTP) phenomena that could potentially destabilize neural circuits.

Indeed, there are very strong homeostatic restrains that tailor synaptic efficiency to the level of activity in the post-synaptic neuron (25).

In line with this model, it has been postulated that enhanced plasticity in dystonia might be the consequence of a disruption of homeostatic plasticity within sensorimotor circuits (26). Following this hypothesis, the homeostatic regulation of cortical plasticity using a neurophysiology protocol combining TMS and transcranial direct current stimulation has been tested and found that patients with FHD have indeed a dys-regulation of synaptic plasticity (27).

This finding was replicated in a subsequent study where the interaction of neuromodulation with use-dependent plasticity induced by repetitive and finger movements has been evaluated.

In healthy subjects, the use of an excitability-enhancing TMS protocol down regulates practice-dependent plasticity, whereas priming with an excitability-depressing protocol increases it (28). This homeostatic modulation was reduced in patients with FHD and was related to the clinical severity of the FHD (29). The presence of abnormal homeostatic control may predispose to the creation of abnormal motor engrams containing redundant information which ultimately may result in dystonia (10).

## Blepharospasm (BPS)

Blepharospasm is a focal dystonia manifested by involuntary eyelid closure (30). The pathophysiology of BPS has some pathophysiological peculiarities in comparison with other focal dystonias since a subtle dopaminergic dysfunction may play an additional permissive role in association with the presence of abnormal plasticity and triggering environmental factors (31).

According to the animal model proposed by Schicatano and associates in rodents, BPS can be obtained inducing a subtle dopamine depletion in the BG combined with a paresis of the orbicularis oculi muscle.

In keeping with this hypothesis, older age, which is associated with a physiological decline of dopaminergic neurotransmission, seems to be a key factor for the development of BPS compared to other types of focal dystonia (32). In addition, in comparison to hemifacial spasm, there is a significant occurrence of ocular symptoms beginning in the year before disease onset and in particular “dry eye” especially when these symptoms developed at an age between 40 and 59 years (33).

Photophobia is another prominent symptom in patients with BPS with light precipitating eyelid spasms (33).

Several hypothesis may account for photophobia in BPS, first of all it has been postulated that central visual neurons may stimulate nociceptor centers in the ophthalmic area of the spinal trigeminal nucleus (33).

Alternatively, it can be hypothesized that the exaggerated pupil constriction induced by bright lights may activate iris nociceptors. Finally, the simplest explanation is that photophobia is produced by intraocular trigeminal nociceptors responding to retinal activity and not by optical nerve (33). This hypothesis is corroborated by the occurrence of photophobia sometimes reported in congenital blind patients (34).

All together these data suggest that central trigeminal sensitization, combined with eyelid spasms, would cause an increased response to intraocular nociceptors, inducing a hypersensitivity to light.

Dry eye or ocular irritation are an important source of trigeminal sensitization in BPS (35) and may explain the linkage between BPS and ocular symptoms (36).

Indirect evidence of increased excitability of the trigeminal system in BPS is provided by the increased plasticity of the blink reflex (37).

This has been demonstrated in BPS by combining a train of electric stimuli delivered to the supraorbital nerve during the R2 (the long latency major response) of the blink reflex (37). This causes a long-lasting augmentation in the R2 amplitude of blink reflex in BPS patients compared to healthy subjects.

The mechanisms underlying the increased plasticity of the blink reflex need to be better clarified. As suggested above, endogenous factors such as a subclinical dopaminergic impairment within BG may produce a brainstem dis-inhibition along with an abnormal gain and plasticity of the blink reflex. This predisposition may trigger a maladaptive plasticity within the brainstem in the presence of environmental factors such as dry eye (31, 37).

### Cervical Dystonia (CD)

Cervical dystonia is characterized by “ involuntary posturing of the head caused by involuntary spasms, jerks, or tremors (or all three combined) and is frequently associated with neck pain” (38).

In patients with CD, several studies point toward an abnormal brainstem excitability that is indexed by an abnormal response to the auditory startle reflex (39), abnormal vestibular and postural reflexes (40), abnormal trigemino–facial reflex (11) and trigemino-sternocleidomastoid reflex (41).

In addition, CD patients have abnormal reciprocal inhibition between agonist and antagonist muscles of the upper limbs at spinal cord level (42, 43).

Transcranial magnetic stimulation has revealed several abnormalities within the primary motor cortex indexed by a reduction of short-interval intracortical inhibition in hand muscles (44) and shortened CSP in the sternocleidomastoid muscles and in cranial muscles in patients with CD (45). Similar to patients with FHD, PAS stimulation revealed enhanced plasticity with loss of topographical specificity in unaffected hand muscles of patients with cranio-cervical dystonia (46).

Cervical dystonia patients may also complain sensory abnormalities such as dry eye sensations or worsening of eyelid closure in bright light. In line with this notion, tactile sensory discrimination is impaired with increased spatial and temporal somatosensory discrimination thresholds in patients with BSP and CD, also in unaffected body regions and in relatives of patients with CD (47–49).

In summary, patients with cranio-cervical dystonia show a reduced inhibition and an abnormal plasticity at various levels of the sensory–motor system.

In addition to the BG, the pontine brainstem (50–53) and cerebellum (54–57) have been implicated in the axial component of dystonia by numerous studies.

In particular, several studies suggest a role of pallidal output neurons to the brainstem in the pathophysiology of dystonia (58, 59).

Anatomical evidence in non-human primate studies has demonstrated that a subset of pallido-thalamic fibers collateralizes to the pedunculopontine nucleus (PPN) and red nucleus (RN) (60). The pallidal projections to PPN are implicated in a wide motor subcortical network involved in the neural control of posture and stabilization (61). These circuits may be well involved in a variety of movement disorders. In particular, while PPN controls muscle tone and posture, the cerebellum, on the other hand, would be involved in balance- and gait-related postural control *via* connections with the RN.

Patients with CD have an altered connectivity between the left ansa lenticularis (AL) and ipsilateral brainstem, and between the right pallidum and ipsilateral brainstem (58). Despite the authors could not establish if these changes were primary or secondary, these data suggest an abnormal connectivity between internal globus pallidus (GPi) and the brainstem which may contribute to the pathophysiology of cervical and possibly generalized dystonia.

## Generalized Dystonia and Deep Brain Stimulation (DBS) Treatment

As discussed in previous sections of this review, several clinical studies point out that abnormalities of plasticity are implicated in the pathophysiology of the different forms of dystonia. Perhaps the best proof is that, in contrast to the almost immediate effects of DBS on the majority of symptoms in Parkinson’s disease, it may take several months to achieve maximum clinical benefit in patients with dystonia. This gradual clinical improvement is paralleled by a similar normalization of several electrophysiological measures of motor inhibition in the brain and spinal cord (19).

These delayed effects of DBS suggest that a process of progressive plasticity and neural reorganization accompanies the long-term effects of GPi DBS (6, 19).

In recent papers of the DBS literature, one can read and re-read about the hypothesis that perhaps DBS through its interference with pathological oscillations in the DBS target areas abolishes the generation of aberrant enhanced neuronal plasticity at early stages of the treatment and thereby allows the system to get rid of engrained abnormal dystonic patterns. While the interference with oscillations is likely to be immediate in the BG, it takes time to erase faulty and re-establish natural motor patterns, indicative of a slow reorganization process (19). The phenomenon observed in that paper suggests that not all parts of the “dystonic electrophysiological signature” consisting of enhanced plasticity, reduced inhibition, and impaired sensory processing are a prerequisite for the existence of clinical dystonia, but, however, are likely to have been important factors during the development and manifestation of dystonia as well as the alleviation of symptoms during stimulation therapy. A study by Barow et al. furthermore suggests that certain dystonic symptoms, more than others, correspond directly to interference with BG oscillations while others are only alleviated after induced modulation of plasticity mechanisms allowed reorganization (62). Among the many facts, one can learn from long term, i.e., many years, DBS-treated patients, one important fact in this context is, that a higher LTP plasticity is correlated with a stability of achieved beneficial motor patterns, meaning that although patients suffer from dystonia their higher LTP seems to serve a beneficial stability of achieved benefit when therapy is withdrawn (63). Remarkably, in some cases of even genetically generated dystonia (DYT1), long-term DBS can lead to a lasting window of improved dystonia after withdrawal of therapy. The time-frame of this window is currently unknown, but has been demonstrated for up to a year. In such a window, although patients suffer from DYT1 dystonia and have a remaining clinical benefit of virtually absent dystonic symptoms, their “dystonic electrophysiological signature” fluctuates without affecting the individual clinical phenotype in that moment of time (64).

In summary, DBS studies in dystonia tell us that the link between electrophysiology pattern and clinical phenotype is not a simple one and might perhaps even have an individual personalized impact.

Similar to focal and multi-focal dystonia patients with generalized dystonia have an increased cortical plasticity. In particular, cortical plasticity is increased following thetaburst stimulation in patients with DYT1 dystonia and sporadic CD but reduced in non-manifesting carriers of the DYT1 gene (65).

An abnormal response to a PAS protocol, such as in FHD, has been reported in patients prior to surgery as compared with healthy individuals (19).

The presence of abnormal plasticity in generalized dystonia is relevant in the comprehension of the therapeutic effects exerted by DBS of the GPi.

As aforementioned, while DBS has an immediate effect on Parkinson’s disease, it may take several months to achieve significant clinical benefit in patients with dystonia. This gradual clinical improvement is associated by a slow normalization of motor inhibition within the brain and spinal cord (19).

These delayed effects of DBS suggest that a significant rearrangement (plastic change) of the cortico-subcortical motor loop may take place after GPi DBS (6, 19).

As said, it has been suggested that the therapeutic effects of DBS are conveyed through its interference with pathological oscillations which in turn would reset aberrant enhanced neuronal plasticity allowing the system to eliminate the engrained abnormal dystonic patterns. While the interference with oscillations is likely to be immediate in the BG, it takes time to fully erase faulty memories and re-establish natural motor patterns, suggesting that plasticity phenomena are at work (19). These data support the idea that not all the “dystonic electrophysiological signatures” such as enhanced plasticity, reduced inhibition, and reduced focality are a prerequisite for the existence of clinical dystonia, but are also important mechanisms in alleviation of symptoms during stimulation therapy.

Interestingly, patients who show larger PAS after-effects are the most likely candidates maintaining the clinical improvement obtained after long-term DBS.

This apparently contradictory effect could be explained considering that patients who have the “highest plasticity” can better re-establish normal motor memories after long term DBS. This paradoxical effect might also explain why patients with enhanced PAS plasticity after years of DBS are the ones who do not worsen when DBS is switched off (63).

In keeping with these considerations, there are cases of sustained relief after DBS discontinuation that may reflect the capacity of DBS to produce long-lasting structural synaptic effects (66).

On the other hand, despite clinical stability after turning DBS off, neurophysiological data may reveal abnormal changes of cortical excitability. This discrepancy between clinical stability and abnormal neurophysiological data, caused by removal of DBS, may be a warning signal indicating a potential risk for a relapse of dystonic symptoms (64).

In this latter perspective, neurophysiological studies may be of value in monitoring the after effects of DBS contributing to individualize treatment in the single patient.

## Anatomo-Functional Models of Dystonia

Despite the fact that there is no adequate neural model that could account for all the symptoms in the different forms of dystonia, anatomical evidence points toward an involvement of the cerebellum and BG.

The role of the BG in dystonia pathophysiology is the most studied in the literature.

A secondary dystonia has been reported in focal lesions of the BG especially when the putamen is damaged (67, 68).

An increase in putamen volume up to 10% has been reported in primary dystonia (69, 70) with fMRI studies showing increased bold signal in the BG (71, 72). Perhaps the best proof of the involvement of BG in dystonia is that the most effective therapeutic surgical target for DBS in dystonia is the internal GPi (73). In addition several animal models of dystonia point toward a role of the striatum (74–78).

Although the involvement of BG is not disputable, the mechanisms producing dystonia have not yet been elucidated.

An involvement of the striatal dopamine and acetylcholine systems emerges from clinical observation since anticholinergic and dopaminergic drugs are still the most used and effective drugs in dystonia (79). On the other hand, acetylcholine agonists and dopaminergic drugs may induce dystonia in humans and primates (80–83).

In keeping with animal data, several pieces of experimental evidence in animal models suggest a pivotal role of striatal cholinergic transmission in the control of voluntary movement and in the pathophysiology of several movement disorders.

More in detail, the D2 receptor agonist Quinpirole produces an abnormal paradoxical excitation of cholinergic interneurons, rather than the expected physiological inhibition in rodent models of DYT1 (84–86).

These data place the imbalance of cholinergic tone within striatal neurons at the core of dystonia pathophysiology (6, 87, 88).

On the other hand, cerebellar lesions can produce different forms of acquired dystonia, particularly CD (5, 89) and several inherited cerebellar ataxias may be associated with dystonic movements (90). In addition, it has been demonstrated that the amount of cerebello-thalamic connectivity may predict the penetrance of DYT1 dystonia (54). These findings may suggest both a compensative and a causative role of the cerebellum in dystonia.

The role of the cerebellum in dystonia pathophysiology is also suggested by several animal studies (91–93).

Although the role of the BG and the cerebellum in dystonia is convincing, on the other hand, this may be a false causal assumption since both structures are strictly connected at cortical and subcortical level.

In line with this thought, animal and human studies support the presence of an extensive multi-synaptic subcortical network connecting cerebellum and BG (55, 94–98). Indeed using a diffusion tensor imaging MRI approach, it has recently been reported, for the first time *in vivo* in humans, that there is presence of extensive connections between BG and cerebellum (95). In particular, in agreement with previous findings of Bostan in monkeys, it has been confirmed in humans that there is existence of a subcortical pathways running between the STN and cerebellar cortex via the pons (94).

In addition, it has also been found evidence for a direct route linking the dentate nucleus to the GPi and to the substantia nigra (95).

The existence of direct dento-pallidal connections is corroborated by a combined magnetoencephalography (MEG)-local field potential (MEG-LFP) study in dystonic patients with DBS to the GPi (99, 100).

In this paper, the authors demonstrated a robust cerebello-pallidal functional coupling in the alpha band which was negatively correlated with clinical symptom severity in patients with cervical or segmental dystonia (99), suggesting a compensatory role of the cerebellum in dystonia.

## Final Considerations

Several lines of evidence suggest that dystonia can be considered to be a network disorder. This view is not entirely new in neurology. In the past, the pathophysiology of movement disorders has been related to a deficit in a single node of the cortico-subcortical loop. However, more recent work at the systems level has revealed how also healthy nodes of the brain at a distance from the primary deficit may react and rearrange themselves in response to the damage. Such plastic reorganization may be either adaptive, compensatory, or maladaptive, worsening the deficit.

As pointed out in the previous section, despite the fact that dystonia has been traditionally linked to a dysfunction within BG a wide array of imaging studies have revealed extensive abnormalities beyond these circuits, including various cortical regions such as the parietal and cingulate cortices, the brainstem, and the cerebellum (4, 5).

In a network model, dystonia can be produced either from a single node dysfunction, from an impairment of multiple nodes or from an abnormal interplay among the nodes (101).

Indeed, the clinical phenomenon of spreading could be related to the possibility of progressive plasticity in remote nodes (6).

At the same time, the delayed effects of DBS suggest a massive but slow rearrangement within remote nodes of the motor loop (19, 102, 103).

Finally, dystonia may arise from an abnormal interplay between nodes (101) such as in dystonias secondary to thalamic lesions (4). Abnormal communication between nodes has been documented with fMRI and EEG. Resting state fMRI, for example, shows reduced connectivity between parietal and dorsal premotor area (104).

Functional connectivity has also been explored in patients with FHD using EEG at rest during a finger tapping task. Using this approach, a significant reduction of beta band connectivity within sensorimotor area in patients with FHD was reported (105). An alteration of oscillatory activity within the motor loop has been demonstrated by DBS studies that have shown an abnormal oscillation in the frequency range of 3–20 Hz, with the low frequency range strictly related to dystonic spasms (106).

These data indicate an abnormal synchronization of brain activity within motor loops which could underlie the abnormal sensory-motor plasticity described above.

It is likely that a misprocessing of sensory feedback combined with an abnormal inhibition within motor circuits may produce a progressive abnormal synchronization and plasticity in local and distant nodes which eventually would result in dystonia (6).

This model could also explain why dystonia may start in one body part (focal dystonia) and then spread to adjacent body regions (multi-segmental dystonia) or even become generalized. Indeed, this delayed time course would be consistent with the spreading of aberrant plasticity across the different nodes (6).

One of the current challenges is to establish whether the alterations across different nodes (spinal cord, somatosensory, BG, cerebellum, cortical) are causative or just compensatory.

These considerations put forward a very complicate puzzle which is different in focal vs generalized dystonia.

## Focal Dystonia Can be Triggered in the Presence of Abnormal Plasticity and Abnormal Inhibition

For instance, in occupational cramps, dystonia may arise in the presence of repetitive training, in oromandibular dystonia after dental surgery and in BPS in association with dry eye. Interestingly, subtle abnormalities of dopamine innervation are another predisposing factor in BPS and this could explain the occurrence in more aged people when there is a physiological decline of dopamine neurotransmission. In generalized dystonia, environmental factors are less important and it is likely that massive structural changes across multiple nodes of the motor loop may play a more relevant role. These structural changes may well explain the delayed effects of DBS in dystonia.

## Author Contributions

AQ: study concept and design, drafting of MS and critical revision. DR: study concept and design, drafting of MS and critical revision.

## Conflict of Interest Statement

The authors declare that the research was conducted in the absence of any commercial or financial relationships that could be construed as a potential conflict of interest. The reviewer FM declared a past co-authorship with one of the authors AQ to the handling Editor.
